# Phage Host Range Expansion Through Directed Evolution on Highly Phage-Resistant Strains of *Klebsiella pneumoniae*

**DOI:** 10.3390/ijms26157597

**Published:** 2025-08-06

**Authors:** Kevin A. Burke, Tracey L. Peters, Olga A. Kirillina, Caitlin D. Urick, Bertran D. Walton, Jordan T. Bird, Nino Mzhavia, Martin O. Georges, Paphavee Lertsethtakarn, Lillian A. Musila, Mikeljon P. Nikolich, Andrey A. Filippov

**Affiliations:** 1 Wound Infections Department, Bacterial Diseases Branch, Center for Infectious Diseases Research, Walter Reed Army Institute of Research, Silver Spring, MD 20910, USA; kevaburke@gmail.com (K.A.B.); olga.a.kirillina.ctr@health.mil (O.A.K.); caitlin.d.urick.ctr@health.mil (C.D.U.); bertran.d.walton.ctr@health.mil (B.D.W.); nino.mzhavia.ctr@health.mil (N.M.); mikeljon.p.nikolich.civ@health.mil (M.P.N.); 2 Department of Entomology, College of Agricultural, Human and Natural Resource Sciences, Washington State University, Pullman, WA 99164, USA; tracey.peters@wsu.edu; 3 Department of Biochemistry and Molecular Biology, University of Arkansas for Medical Sciences, Little Rock, AR 72205, USA; jordantobybird@gmail.com; 4 Department of Emerging Infectious Diseases, Walter Reed Army Institute of Research-Africa, Nairobi P.O. Box 606-00621, Kenya; martin.georges@usamru-k.org (M.O.G.);; 5 Department of Bacterial and Parasitic Diseases, Walter Reed Army Institute of Research-Armed Forces Research Institute of Medical Sciences, Bangkok 10400, Thailand; paphaveel.fsn@afrims.org

**Keywords:** *Klebsiella pneumoniae*, phage training, Appelmans protocol, *Jiaodavirus*, host range expansion, defined mutations and recombination events, phage cocktail improvement

## Abstract

Multidrug-resistant (MDR) strains of *Klebsiella pneumoniae* present an acute threat as they continue to disseminate globally. Phage therapy has shown promise as a powerful approach to combat MDR infections, but narrow phage host ranges make development of broad acting therapeutics more challenging. The goal of this effort was to use in vitro directed evolution (the “Appelmans protocol”) to isolate *K. pneumoniae* phages with broader host ranges for improved therapeutic cocktails. Five myophages in the genus *Jiaodavirus* (family *Straboviridae*) with complementary activity were mixed and passaged against a panel of 11 bacterial strains including a permissive host and phage-resistant clinical isolates. Following multiple rounds of training, we collected phage variants displaying altered specificity or expanded host ranges compared with parental phages when tested against a 100 strain diversity panel of *K. pneumoniae*. Some phage variants gained the ability to lyse previously phage-resistant strains but lost activity towards previously phage-susceptible strains, while several variants had expanded activity. Whole-genome sequencing identified mutations and recombination events impacting genes associated with host tropism including tail fiber genes that most likely underlie the observed changes in host ranges. Evolved phages with broader activity are promising candidates for improved *K. pneumoniae* therapeutic phage cocktails.

## 1. Introduction

*Klebsiella pneumoniae* is an important human pathogen that tends to acquire and spread drug resistance and causes various hospital-acquired infections including lung, urinary tract, bloodstream, brain, wound, and surgical site infections. Carbapenem-resistant *K. pneumoniae* is categorized as an urgent public health threat by the Centers for Disease Control and Prevention [1,2,3]. Multidrug-resistant (MDR) infections caused by *K. pneumoniae* strains have been reported from multiple hospitals on all continents, from developing and developed countries alike, meaning they are a global challenge [4,5,6,7,8,9,10,11]. Some high-risk MDR *K. pneumoniae* lineages, especially hypervirulent strains, cause infections with high mortality that can reach 50% [12,13,14,15,16,17].

The need for effective alternative antibacterials is great, and bacteriophage (phage) therapy presents a promising avenue for the treatment of MDR *K. pneumoniae* infections, often in combination with standard-of-care antibiotics. Phages have been employed to successfully treat *K. pneumoniae* infections both in animal models and in human compassionate use cases [18,19,20,21,22,23,24]. Our team is focused on the development of broadly active, durable phage cocktails for utilization in multiple clinical cases against diverse strains. This is a difficult task for *K. pneumoniae* because of the high diversity of its surface factors, particularly capsule types [25], and the narrow host ranges of *Klebsiella* phages, which typically vary from 1% to 31%, even when tested against relatively small strain panels or those with limited or unspecified diversity [26,27,28,29,30]. We screened *Klebsiella* phages against a 100 strain diversity panel of mostly MDR *K. pneumoniae* clinical isolates that includes 94 different sequence types, 54 KL serotypes, and 11 OL serotypes [31]. Given this high diversity, even a library of 151 *Klebsiella* phages harvested on four continents and belonging to 25 genera was unable to cover >63% of the *K. pneumoniae* panel.

We, therefore, employed an in vitro directed evolution strategy (also known as phage training or host adaptation) using a general method called the Appelmans protocol [23,32,33], to generate phage variants with expanded coverage against the highly diverse panel of clinical *K. pneumoniae* isolates. This approach yielded nine *Jiaodavirus* phage variants with altered specificity and genome sequences, including three isolates with expanded host ranges, one of which provided dramatically expanded activity in iterative phage cocktail development. The findings of this study demonstrate the potential of phage training in the rational design of therapeutic phage cocktails to overcome the limitations of narrow lytic spectra of cocktail components within a target bacterial species, particularly with species such as *K. pneumoniae* that typically have phages with narrower host ranges.

## 2. Results

### 2.1. Training Led to Phage Variants with Altered Lytic Spectra

Eleven *K. pneumoniae* strains used for phage training (Table 1) were selected based on their phage resistance, antibiotic resistance, and diversity, representing 11 sequence types, 10 capsule (KL) serotypes, and five O-antigen (OL) serotypes. A phage-susceptible strain, MRSN 414780, was used for the general propagation of training lysates and maintaining phage titers during training. The five training phages (Table 2) included vB_Kpn11382-KEN22 (KEN22), vB_Kpn529046-KEN25-1 (KEN25-1), vB_Kpn529046-KEN25-2 (KEN25-2), vB_Kpn529046-KEN37 (KEN37), and vB_Kpn529046-KEN39 (KEN39). Their genomes were previously published [34]. They belong to the genus *Jiaodavirus* in the myophage family *Straboviridae,* whose members are reported to have relatively broad host ranges, quick lysis times, and efficacy at lower multiplicities of infection [35]. These phages were selected based on their genetic similarity, which is important to help facilitate recombination, a likely major driver of change in training experiments [33]. Another criterion for selection was complementarity in the host range coverage to achieve expansion of activity.

**Figure 1:**
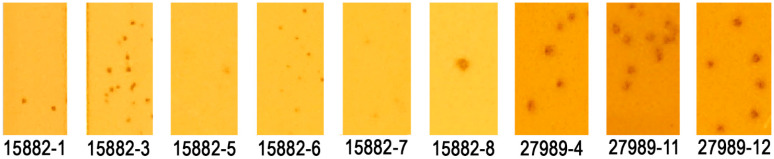
Plaque morphology of phage isolates following 10 rounds of training on phage-resistant strains MRSN 15882 and MRSN 27989.

Following 10 rounds of phage training (see Materials and Methods), we were able to isolate multiple phage variants infecting highly phage-resistant strains that were initially resistant to the parental phages. Phage plaques were collected from and purified on *K. pneumoniae* strains MRSN 15687, MRSN 15882, MRSN 27989, and MRSN 681054, but some of these variants were seemingly unstable and were either lost before being purified or recalcitrant to propagation to workable phage titers. Nine relatively stable phage variants were isolated on highly resistant strains MRSN 15882 and MRSN 27989; six trained variants were isolated and purified on MRSN 15882 and three on MRSN 27989 (Figure 1).

**Figure 2:**
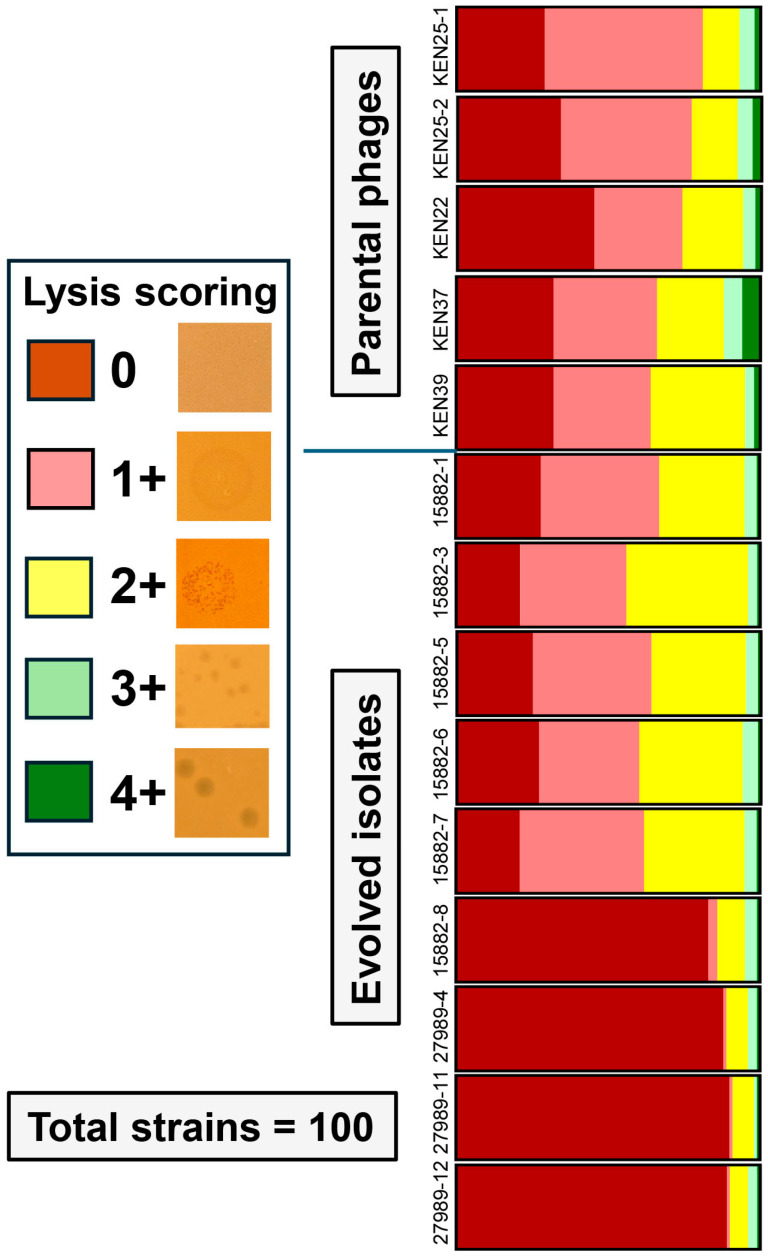
Host ranges of trained and parental phages on the 100 strain *K. pneumoniae* diversity panel following 10 rounds of training. Lysis scoring was performed using a qualitative scoring system based on plaque formation and quality, which is presented in Table 4.

After three rounds of plaque isolation, nine evolved phages were tested for host ranges against the panel of 100 highly diverse *K. pneumoniae* clinical isolates [31]. Additionally, parental phages KEN22, KEN25-1, KEN25-2, KEN37, and KEN39 were plated on the panel at the same time. All these new phage variants displayed altered lytic spectra and three of them, 15882-3, 15882-6, and 15882-7, demonstrated expanded host ranges of 44%, 40%, and 38%, respectively (Table 3 and Figure 2).

Compared with the best-performing parental phage, KEN39, which was active against 36% of strains (Table 3), this represented expansion rates of 8%, 4%, or 2%, respectively. No other trained phages revealed expanded host ranges. Isolates 15882-1 and 15882-5 showed 33% and 36% activity (roughly equivalent to the parental phages). Finally, phage variants 15882-8, 27989-4, 27989-11 and 27989-12 showed host ranges narrowed by 14%, 11%, 9%, and 10%, respectively (see Table 3 and Figure 2). All phages with expanded host ranges showed productive infection and plaque formation on previously resistant strains, but the quality of lysis was limited at a score of 2+ (Table 4). Even evolved phages with narrowed or equivalent host ranges were capable of lysing the highly phage-resistant strains MRSN 15882 and MRSN 27989.

### 2.2. Serial Propagation on a Phage-Susceptible K. pneumoniae Strain Resulted in Host Range Changes

**Figure 3:**
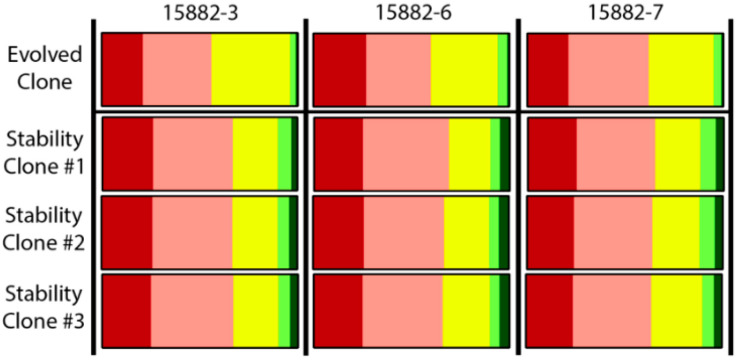
Host ranges of phage clones collected after serial propagation on the permissive host strain *K. pneumoniae* MRSN 414780. These clones were compared to the originally evolved trained phages by plating on the 100 strain diversity panel. Phage activity color coding is described in Table 4 and Figure 2.

Purified evolved phages were assessed for the stability of the acquired host range changes by passaging against the phage-susceptible strain MRSN 414780. Following five serial propagations on MRSN 414780, progeny phage clones were collected and their host ranges were determined and compared to their parental trained phage variants (Figure 3). Three clones were collected from each lysate, 15882-3, 15882-6, and 15882-7. All of the progeny clones displayed some narrowing of the overall host range due to tapered 2+ activity shown in yellow. However, the proportion of strains lysed with full activity (scores 3+ and 4+; see Table 4 and Figure 3) increased in all the progeny clones. For example, the originally evolved isolates lysed with 4+ activity only one strain, MRSN 414780, in contrast to four, five, or four strains for the 15882-3, 15882-6, and 15882-7 progeny clones, respectively (Figure 3). The strains lysed with higher-level activity primarily expressed O-antigen types O3b and O1v1. Therefore, to maintain stability, we intend to store phage stocks with expanded host ranges propagated on the phage-resistant strain MRSN 15882 and to use the same strain for propagation of the evolved phages in the future.

### 2.3. A Broad Host Range Cocktail Containing an Evolved Phage

**Figure 4:**
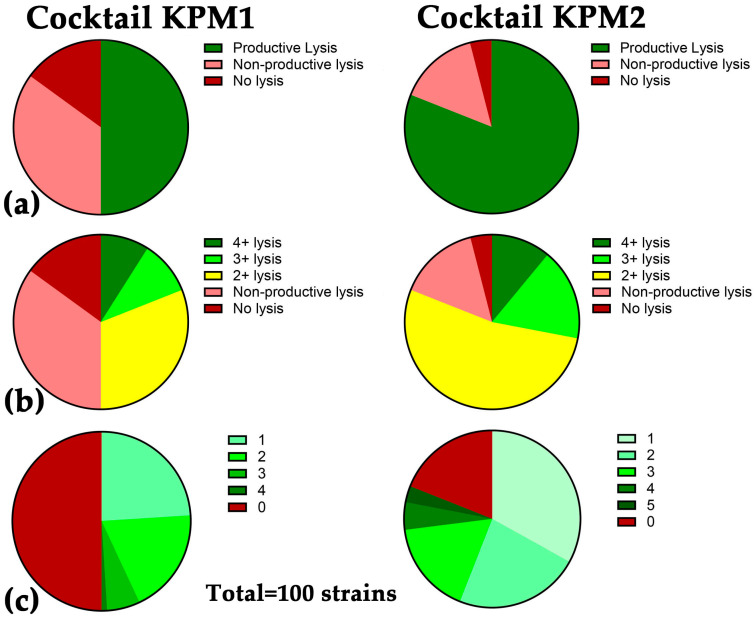
Comparative activity of phage cocktails WRAIR_KPM1 and WRAIR_KPM2: (**a**) KPM1 covers 50% of strains in the 100 strain *K. pneumoniae* diversity panel, while KPM2 is active against 81% of the strains; (**b**) overall higher lytic activity of KPM2 phages; (**c**) greater numbers of strains lysed by two or more KPM2 components compared to KPM1.

The ultimate goal of this study was to isolate phage variants with expanded host ranges that could be incorporated in a therapeutic phage cocktail for improved formulations. Our previous best-performing cocktail targeting *K. pneumoniae*, WRAIR_KPM1 (KPM1), included five phages, KEN39, EKq1, EKq2, AFR4, and KEN42, which collectively lyse 50% of the diversity panel strains. The addition of the trained phage 15882-3 to KPM1 expanded its activity by 21%. We developed an improved version of KPM1, designated WRAIR_KPM2 (KPM2) (Table 5), comprising the core (KPM1) phages along with the evolved phage 15882-3 and recently isolated natural phage KEN1821. This cocktail covered 81% of the diversity panel (Table 5, Figure 4a). KPM2 also outperformed KPM1 in its quality of lysis, with more robust plaque formation (Figure 4b) and more strains lysed by two or more phages (Figure 4c).

### 2.4. Sequence Analysis of Trained Phages Reveals Multiple Recombination Events and Accumulation of Mutations

**Figure 5:**
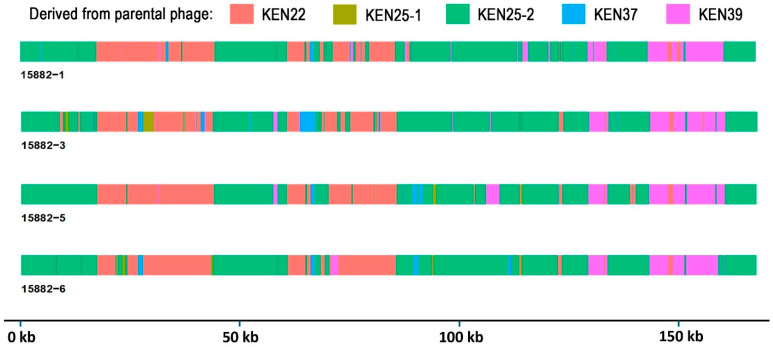
Genome maps of four recombinant *Jiaodavirus* phages isolated from phage training experiments. Segments derived from each parental phage are shown in different colors. A variant analysis was conducted using NucDiff; figure was generated using gggenomes in RStudio.

Four evolved phages were selected for variant and recombination analyses: 15882-1, 15882-3, 15882-5, and 15882-6. Since the five input parental phages shared a nucleotide identity range of ~90–94%, recombination was expected. All four isolates were found to be recombinant phages, primarily derived from parental phages KEN22 and KEN25-2 (~94% identity, see Table 6). Multiple recombination events were detected (Figure 5) in genes that encode for RNA and nucleotide metabolism, baseplate, and tail proteins. Genes that are likely involved in host range expansion and phage–host interactions with significant recombination events or polymorphisms resulting in unique variation from parents are described below.

### 2.5. Recombinant Events Resulted in Few Nonsynonymous Mutations Compared to Parental Phages

Although one or more recombination events were identified in genes involved in phage–host interactions, these recombination events did not frequently result in amino acid substitutions unique from parental genomes (Table S1). Genes that housed amino acid substitutions unique from parents include those encoding an anti-sigma factor gene and the hinge connector protein of the long tail fiber. The anti-sigma factor gene (reference genome KEN22, NCBI accession PP723050, locus WZPMBQFE_CDS0086) is homologous to gp49 of T4, a recombination endonuclease VII, which is expressed at early and late stages of T4 infection and is involved in mismatch repair, the resolution of branched DNA, and DNA packaging [37]. The hinge connector protein (WZPMBQFE_CDS0082) is homologous to gp34 of phage T4, which is a homo-trimer that forms the proximal half-fiber, with the N-terminal end binding to the baseplate and the C-terminal end being involved in the hinge between the proximal and distal half fibers [38]. We used two machine learning tools for identification of potential depolymerase sequences, and the hinge connector protein was scored at 100% by DepoScope [39] v1.0 for all parental phages, except KEN37, but was scored only at 42–60% by PhageDPO [40].

Recombination events were detected in a baseplate hub subunit and tail length protein gene ((WZPMBQFE_CDS0027) in three evolved phages (except 15882-1, which shared 100% nucleotide identity in this gene to KEN22), with segments derived from KEN22 and KEN25-2. Variant 15882-6 had multiple recombination events in this gene, but surprisingly had 100% identity at the amino acid level to this gene in KEN25-2. Furthermore, 15882-3 and 15882-5 shared 100% identity in this gene and had two amino acid changes in the C-terminal compared to KEN22 (residues 555 His -> Tyr, and 559 Met -> Thr); these amino acid substitutions reflected those found in parental phage KEN25-2. This gene showed weak homology to the tape measure protein of phage DT57C [41]. Preliminary structural predictions and an analysis using AlphaFold 3 [42] and the RCSB pairwise structure alignment tool [43] for phages KEN22, KEN25-2, and 15882-3 suggest that these structures may differ in their length.

The tail fiber protein gene (WZPMBQFE_CDS0083) showed recombination between KEN22 and KEN25-2 in all four evolved phage variants, whereby 15882-1 and 15882-6 shared 100% identity, while 15882-3 and 15882-5 shared 100% identity and also included a recombination region from KEN39 at the N-terminal. Despite recombination events detected in this gene, no unique amino acid changes were identified compared to the parental phages. This gene had regions of homology to the putative tail fiber protein of *Ralstonia* phage GP4 [44] (probability 98.63%, e-value 2.6 × 10^−6^), the L-shaped tail fiber assembly of phage T5 [45] (probability 98.12–98.35%, e-value 2.1 × 10^−5^–1.2 × 10^−6^), and the phage T4 proximal long tail fiber gp34 [38] (probability 91.96%, e-value 7.4). This gene is located immediately downstream of the hinge connector protein gene and was scored by PhageDPO [40] as a depolymerase at 98% for KEN22, 99% for KEN25-2, and 95% for KEN39. DepoScope [39] scored this gene at 90% for KEN25-2, suggesting a high probability of depolymerase activity for this tail fiber protein. Preliminary structural predictions and an analysis for KEN22, KEN25-2, KEN39, 15882-3, and 15882-6 tail fibers showed that the recombinant tail fiber proteins from trained phages 15882-3 and 15882-6 have predicted structures unique from the parents.

Recombination was detected between KEN22 and KEN39 in a gene annotated as a baseplate hub subunit and tail lysozyme for isolate 15882-1 (WZPMBQFE_CDS0279), which is homologous to gp5 of the T4 phage [46]. This results in two amino acid changes compared to KEN22 at residues 494 (Asn -> Asp) and 516 (Ser -> Asp), which corresponds to the residues present in KEN39; however, no unique amino acid substitutions were identified.

A genetic analysis of phage clones collected after serial propagation on the permissive host strain *K. pneumoniae* MRSN 414780 showed that only two clones had additional mutations compared to the original trained phage they were derived from. 15882-3-2 had two mutations, one of which was an SNP in an intergenic region between genes that encode for lysis inhibition protein and a head morphogenesis protein (see Table S1) and a nonsynonymous mutation in a thioredoxin domain-containing protein. Thioredoxin domain-containing proteins can influence the phage DNA replication efficiency by interacting with the bacterial host machinery, such as DNA polymerase [47]. Phage clone 15882-6-4 showed one additional mutation in a gene that encodes a tail collar fiber protein (reference genome KEN25-2, NCBI accession PP723048, locus BXUKSMXV_CDS0283). This gene showed homology (probability 100%, e-value 2.1 × 10^−32^) to the phage T4 gene encoding gp12, a short tail fiber protein, which is part of the tail fiber network forming a spring-like mechanism that extends upon interaction with a suitable host cell, with the N-terminal domain of gp12 orienting towards the host cell surface to bind to a receptor [48]. This gene was scored by PhageDPO [40] as a depolymerase at 85% for KEN25-2 and 86% for phage 15882-6-4.

## 3. Discussion

*K. pneumoniae* is a challenging target species for the development of broad-range phage cocktail therapeutics. In large part, this is because of the tremendous diversity of cell surface targets, especially capsule and lipopolysaccharide (LPS) types, which are known to serve as phage receptors or otherwise influence phage susceptibility [25,49,50]. For example, the 100 strain diversity panel of *K. pneumoniae* clinical isolates used in this study was created based on multilocus sequence typing (MLST), and includes 94 sequence types (STs), 54 capsule types, and 11 O-antigen serotypes [31]. The strains in this panel were isolated between 2003 and 2020 in multiple military hospitals in North and South America, Europe, Asia, and Africa, from blood, wound, urine, respiratory, perianal specimens, and environmental swabs.

The *Klebsiella* phage host range has been correlated to ST and capsule types, which themselves are correlated with the 92% probability of a phage lytic against one strain of certain capsule type being lytic against another strain of the same capsule type [51]. Phages need to overcome the thick capsule layer to reach the cell surface. This often requires the phage to encode depolymerase enzymes that can degrade the capsule layer. *Klebsiella* phages encoding one or a few depolymerases are more likely to have a narrow host range in comparison to phages that encode multiple or divergent depolymerases [52,53,54,55]. This makes a broadly active *Klebsiella* phage a relatively rare phenomenon as it requires a phage encoding many copies of divergently active depolymerases. One such rare *K. pneumoniae* phage possessed 11 distinct depolymerases, enabling the lysis of 10 distinct capsule types [56]. Interestingly, phages isolated on capsule-deficient mutants of *K. pneumoniae* have shown relatively broad host ranges [57].

The diversity in capsule and LPS types results in narrow specificity of most *Klebsiella* phages that usually cover only 1–31% of the tested *K. pneumoniae* strains [26,27,28,29,30]. Moreover, the panels of strains used for host range testing in these published studies were small or limited in diversity, or diversity was not specified. Only a few reported *Klebsiella* phages have shown broader activity, and there was no evidence for any of them to be effective against diversity strain panels. For example, phages KP34 [58] and vB_KpnM_M1 [23] covered 42/101 (42%) and 76/121 (63%) of *K. pneumoniae* strains, respectively, but the diversity of the strains was not specified. Phage Kpp95 was lytic against 47% of 108 *K. pneumoniae* isolates from the same hospital [59]. Phages vB_Klp_3 and vB_Klp_4 were active against 76% of 73 *Klebsiella* spp. isolates from University Hospitals of Leicester (UK), but only 26% of 50 Georgian isolates [60]. Finally, phages P545 and P546 showed as broad lytic spectrum as 96%, but they were evaluated against 54 *K. pneumoniae* strains isolated from the same hospital, 48 of which (89%) belonged to the same sequence type, ST11 [61]. Given that the 100 strain *K. pneumoniae* panel used in this work is highly diverse [31], it should not be surprising that the majority in our 151 phage library had host ranges varying between 1% and 7%, with the broadest activity of 36% in phages KEN39 and KEN1821 (Table 2). Therefore, the goal of this work was to use directed evolution to obtain *K. pneumoniae* phages with broader host ranges and employ them for improving therapeutic phage cocktails.

Phage directed evolution (training, host adaptation) can result in host range expansion [62]. Several researchers have successfully used for host range expansion the Appelmans protocol, with the incubation of serial phage dilutions with bacterial cultures and lysis monitoring in liquid media first described in 1921 [63]. Using both permissive and phage-resistant bacterial strains, this method has been employed to expand the lytic spectra of phages specific for *Staphylococcus aureus* [64,65,66,67], *Pseudomonas aeruginosa* [33,68,69], *Enterococcus faecium* [70], *Acinetobacter baumannii* [71], and *Listeria monocytogenes* [72], as well as *Escherichia coli* [73,74], *Streptococcus* spp. and *Enterococcus* spp. isolates [74] from urinary tract infections (UTI). *Klebsiella* phage vB_KpnM_M1, which belongs to the genus *Slopekvirus,* was adapted by the Appelmans protocol to pan-drug-resistant *K. pneumoniae* isolates from a polytrauma patient; this led to a reduced incidence of phage resistance and higher efficacy of phage treatment [23]. Compassionate use therapy with trained phages was also effective in 6/9 patients with UTIs caused by *E. coli, Streptococcus* spp., and *Enterococcus* spp. [74].

Using two phages that belong to the same genus resulted in recombinant phages with expanded activity against *P. aeruginosa* [33] and *L. monocytogenes* [72]. In this work, we utilized five myophages of the genus *Jiaodavirus* (Table 2). This group of phages is characterized by a broad host range, quick lysis time, and efficacy at lower multiplicities of infection [35]. These five phages were trained against 11 diverse MDR and XDR *K. pneumoniae* clinical isolates, including one phage-susceptible and 10 phage-resistant strains (Table 1 and Table 2). Nine evolved phage variants with altered host ranges were isolated, which were capable of lysing previously phage-resistant strains. Four of these variants demonstrated host range expansion of up to 8% compared to the parental phage KEN39 with the broadest activity (Table 3, Figure 2). Finding few unique substitutions (Table S1) and multiple recombination events (Figure 5) in the evolved phages suggests that recombination plays a significant role in host range alteration and expansion in *Jiaodavirus* phages, likely due to the generation of chimeric proteins involved in phage–host interactions, such as those observed in the tail fiber protein of trained phages 15882-3 and 15882-6. However, unique mutations in the gene encoding the hinge connector protein of long tail fibers were identified in three of the four genomically characterized evolved phages; thus, this gene may also be significant for host range expansion.

Although the parallel host presentation approach employed here resulted in evolved phages with an expanded host range, when evolved phage isolates were serially passaged on a single host, some progeny clones displayed a decrease in host range (15882-3 and 15882-6). As observed previously, parallel host presentation can support both generalist and specialist phages, while serially passaging a phage on a single host can select for specialist phages [75]. The mutations identified in progeny clones 15882-3-2 and 15882-6-4 coupled with their phenotypic data indicate that these phages may have evolved to become more efficient in replicating in and/or lysis of select host strains.

Several groups of scientists recently reported that the Appelmans procedures were thwarted by prophage induction; expanded phage activity occurred not because of mutations or recombination events in input phages but because of induced prophages. For example, an attempt to train lytic *A. baumannii* phages P115, P711, and P577 resulted in the isolation of four phages with broader host ranges that were recombinant derivatives of prophages from development bacterial strains [76]. A study with the Appelmans method employing two *Yuavirus* and one *Detrevirus* phages detected a *Casadabanvirus* prophage that was induced from the *P. aeruginosa* chromosome and caused host range expansion [77]. The use of three lytic *Przondovirus* phages in a training experiment on *K. pneumoniae* enabled the isolation of temperate phage vB_KpnS-KpLi5, which expanded the activity of the input phages [78]. Such temperate phages derived from training procedures cannot be used for phage therapy. This kind of prophage induction that expands lytic spectra of input phages was not observed in the work described herein.

Phage training is currently recommended for use for the improvement of therapeutic phage cocktails [32,79]. Our team is developing durable fixed phage cocktails against MDR ESKAPE pathogens, including *K. pneumoniae* [80]. The first iteration of the *K. pneumoniae* phage cocktail, KPM1 (Table 5), consisted of five natural phage isolates and covered 50/100 (50%) of the strains in the high-diversity panel [31], including 48 different STs, 32 KL serotypes, and 9 OL serotypes. Most of the STs are global epidemic, MDR, and XDR lineages, e.g., ST11, ST14, ST15, ST20, ST37, ST45, ST101, ST107, ST147, ST258, ST322, ST336, ST340, ST394, and ST512 [2,4,5,9,10,11,81]. However, 50% coverage is not enough for an off-the-shelf phage cocktail. The addition of trained phage 15882-3 to this cocktail expanded its activity by 21% and allowed it to cover two additional XDR strains of global epidemic lineage ST147 and 19 sequence types resistant to KPM1 phages (ST1, ST16, ST39, ST44, ST111, ST307, ST348, ST391, ST661, ST686, ST1583, ST1686, ST1787, ST1838, ST2279, ST2623, ST5445, ST5448, and ST5449). Of them, ST307 and ST16 are global high-risk clones often associated with the XDR phenotype, while ST1, ST39, ST111, ST348, and ST661 are spread in different countries and tend to grow in drug resistance [5,6,7,81,82]. After the incorporation of recently isolated wild-type phage KEN1821, the 7 phage cocktail KPM2 had a broad host range of 81% (Table 5, Figure 4a). KPM2 covered 78/94 STs, 45/54 KL serotypes, and representatives of all 11 OL serotypes in the diversity strain panel. This improved cocktail also outperformed KPM1 in its quality of lysis, with more robust plaque formation (Figure 4b) and higher numbers of strains lysed by two or more phages (Figure 4c).

To conclude, the use of five *Jiaodavirus* phages in the Appelmans procedure against broadly phage-resistant strains allowed for the isolation of progeny phage variants with altered and expanded host ranges. Genome recombination played a significant role in the alteration and expansion of phage lytic spectra. The incorporation of a trained phage into iterative phage cocktail design dramatically expanded host range against *K. pneumoniae* global diversity represented in a 100 strain panel comprising 94 sequence types.

## 4. Materials and Methods

### 4.1. Bacterial Strains, Phages, Growth, and Storage Conditions

In addition to 11 *K. pneumoniae strains* used for phage training (Table 1), strains MRSN 3619, MRSN 11382, and MRSN 529046, and *Klebsiella quasipneumoniae* strain MRSN 829456, were utilized for the propagation of phages before training (Table 2). Bacterial cultures were grown in Heart Infusion Broth (HIB, Becton, Dickinson and Company, Franklin Lakes, NJ, USA) at 37 °C, with shaking at 120 rpm, or on HIB agar. Fresh overnight cultures were prepared for each experiment. Phages used in this work are listed in Table 2. They included five training phages (KEN22, KEN25-1, KEN25-2, KEN37, and KEN39) and five phages used for the development of phage cocktails broadly active against MDR *K. pneumoniae* isolates (KEN42, KEN1821, AFR4, EKq1, and EKq2). For propagation, a concentrated phage stock was added to bacterial culture at mid-log phase in HIB with 2 mM of CaCl_2_ and 10 mM of MgSO_4_ (HIB-CM) at a multiplicity of infection of ca. 0.01, then allowed to incubate for 4–6 h until the culture was cleared. Debris was pelleted, and the phage lysate was sterilized by passing it through a 0.22 µm syringe filter. Filtered phage lysates were used as the initial inocula for phage training. Phage lysates were stored at +4 °C, protected from light.

### 4.2. The Appelmans Training

Phage training using the Appelmans protocol was conducted as described previously [33], with modifications. The initial phage inoculum consisted of a 1:1:1:1:1 mix of KEN22, KEN25-1, KEN25-2, KEN37, and KEN39 at a final concentration of 1 × 10^10^ PFU/mL (2 × 10^9^ PFU/mL per each phage). One hundred and ten microliters of the phage mix was added to the first well of a 96-well plate, titrated 10-fold with 90 µL of SM buffer (Alpha Teknova, Inc., Hollister, CA, USA) down the columns of the plate to the seventh well, and 100 µL of 2 × HIB-CM was added to each well. The cultures of *K. pneumoniae* phage-resistant clinical isolates (Table 1) were grown in 1 × HIB-CM for 16 h at 37 °C, with shaking at 180 rpm. Titrated phage mixes were inoculated with 2 µL of the overnight bacterial cultures containing approximately 1.5 × 10^6^ CFU, with one strain per column. The multiplicities of phage infection varied in the seven plate wells with the titrated phage mix roughly from 700 to 0.0007. An uninfected bacterial control (100 µL of SM buffer, 100 µL of 2 × HIB-CM, and 2 µL of overnight bacterial culture) was maintained for all strains used. The assembled plate was incubated overnight at 37 °C with shaking, and the OD at 600 nm was visualized in a microplate reader (SpectraMax Plus 384, Molecular Devices, San Jose, CA, USA). Wells that showed reductions in OD relative to the uninfected control were tracked. The two most diluted lysates showing OD reductions were collected to select for the best performing possible recombinant or mutant phages. If no reduction in OD was observed, the two least diluted samples were collected. All collected lysates were pooled together, filter-sterilized, and stored at 4 °C for subsequent use. Filtered lysates from round 1 of phage training were titrated in SM buffer, diluted with 2 × HIB-CM, and inoculated with *K. pneumoniae* cultures as described above (round 2), and the procedure was repeated for all subsequent rounds of training. To assess for activity on phage-resistant strains, pooled lysates were titrated in SM buffer to 10^−7^ and plated on the strains used in the phage training. Plaque formation was assessed, and plaques were collected for the follow-up analysis.

### 4.3. Purification of Collected Trained Phage Variants

Phages isolated on previously resistant strains were collected by picking plaques on double-layer HIB agar (1.5%/0.7%) and transferring them to 500 µL of sterile SM buffer. Fifty microliters of chloroform was added to assist in destroying any remaining cells and releasing phages still inside. After a 15 min incubation at room temperature, the plaque suspension was centrifuged at 5000× *g* for 5 min. The aqueous phase was collected and filter-sterilized. The process of phage plating, single plaque isolation, and filter sterilization was repeated three times, and the phage purity was then assessed by confirming uniform plaque morphology.

### 4.4. Host Range Determination

The host range was determined by assessing lysis against the 100 strain *K. pneumoniae* diversity panel [31] of mostly MDR clinical isolates as described previously [66], with minor modifications. Briefly, overnight cultures of bacterial strains were grown in HIB. Ten-fold serial dilutions of the tested phages were prepared in a sterile flat bottom 96-well plate. An aliquot (2 μL) of each phage dilution, ranging from 10^−1^ to 10^−8^, was spotted using a multichannel pipette on 0.7% HIB agar overlay infused with *K. pneumoniae* culture and incubated overnight at 37 °C. The following day, the quality of lysis was assessed.

### 4.5. Assessment of Stability of Host Range Expansion

The stability of observed changes in lytic spectra was assessed by conducting five serial propagations on a single-phage-susceptible strain, *K. pneumoniae* MRSN 414780. This was followed by the re-isolation and purification of clones and re-assessment of host ranges as described in Section 4.4, which was compared with the originally isolated trained phage clones.

### 4.6. DNA Isolation, Library Preparation, Sequencing, and Genome Assembly

Phages were propagated on a corresponding host strain, and their DNA was extracted as described [83] using the QIAamp DNA Mini Kit (Qiagen, Germantown, MD, USA). Sequencing libraries were constructed using the KAPA HyperPlus Kit (Roche Diagnostics, Indianapolis, IN, USA) and sequenced on an Illumina MiSeq (Illumina, Inc., San Diego, CA, USA) with a 600 cycle MiSeq Reagent Kit v3 that produced 300 bp paired-end reads. The quality of the reads was assessed, and then trimmed using Fastp [84] v0.22.0. Genomes were then de-novo-assembled from the trimmed reads using Unicycler [85] v0.5.0 using both paired and unpaired reads. Where necessary, trimmed read datasets were subsampled using seqtk (https://github.com/lh3/seqtk) (accessed on 7 December 2024) v1.4 to achieve ~100× of expected genomes prior to genome assembly. Assembly statistics and average coverage of assembled genomes were determined using BBmap (https://sourceforge.net/projects/bbmap) (accessed on 9 December 2024) v38.9 and SAMtools [86] v1.13.

### 4.7. Genome Annotation

The termini of each assembled phage genome were identified using PhageTerm [87] v1.0.12. Phage protein coding sequences (CDSs) were annotated using the Pharokka pipeline [88,89,90,91,92,93,94,95,96,97,98,99]. Pharokka [88] integrates predicted coding sequences (CDS) from PHANOTATE [89] with functional annotations generated by matching each CDS to the PHROG [90], VFDB [91], and CARD [92] databases (accessed 10 December 2024) using MMseqs2 [93] v13.45111 and PyHMMER [94] v0.11.0. The tRNAs and tmRNAs were predicted with tRNAscan-SE 2.0 [95] v2.0 and ARAGORN [96] v1.2.41, respectively, and CRISPRs were predicted with CRT [97]. Phage contigs were also matched to their closest hit in the INPHARED database [98] using mash [99] v2.3. A depolymerase analysis was conducted using DepoScope [39] v1.0 and PhageDPO [40] using default settings.

### 4.8. Genome Variation Analysis

Structural variations, single-nucleotide polymorphisms (SNPs), insertions, and deletions (indels) were identified between phage mutant genomes and their respective parental strains using NucDiff [100] v2.0.3. NucDiff aligns input genomes and detects variants including substitutions, insertions, deletions, inversions, and translocations. The R package gggenome [101] v1.0.0 was used to visualize genomic variations in RStudio (2024.09.0 + 375). Aligned genomes were provided as inputs to generate figures depicting SNPs, indels, and structural rearrangements. Default gggenome plotting parameters were used, except for varying figure sizes and color schemes. Protein homology searches were performed using HHpred [102] v3.3.0 and Phyre2 [103] v2.1.

## Figures and Tables

**Table 1:** Clinical isolates of *K. pneumoniae* used for phage training.

Strain	Sample Type	Antibiotic Susceptibility ^1^	Phage Susceptibility ^2^	Sequence Type	KL Serotype	OL Serotype
MRSN 4759	Urine	MDR	XPR	ST37	KL38	O3b
MRSN 6778	Urine	MDR	XPR	ST1842	KL3	O2v2
MRSN 15687	Urine	MDR	XPR	ST5446	KL62	O2v1
MRSN 15882	Perianal	MDR	XPR	ST1686	KL113	O1v1
MRSN 22232	Respiratory	XDR	XPR	ST405	KL151	O4
MRSN 27989	Wound	MDR	XPR	ST2279	KL3	O2v2
MRSN 479404	Wound	XDR	XPR	ST16	KL51	O3b
MRSN 511348	Unknown	XDR	XPR	ST14	KL2	O1v1
MRSN 614201	Environ.	MDR	PPR	ST1838	KL14	O3b
MRSN 681054	Urine	XDR	PPR	ST340	KL15	O4
MRSN 414780	Urine	XDR	PS	ST323	KL21	O3b

Table displays antibiotic and phage susceptibility of strains used in the training. Strains were assessed by reviewing antibiotic MIC data [31] and phage susceptibility data. ^1^ XDR, extensively drug-resistant. ^2^ XPR, extensively phage-resistant: these eight strains were resistant to all phages used for training. Five of them were fully resistant (no plaque formation, no lysis spots), and three of them (MRSN 15687, MRSN 27989, and MRSN 479404) showed lysis from without by these phages (no plaque formation, slight confluent lysis zones only in high titers). XPR strains were susceptible only to 0.7–3% of phages from the 151 phage panel available in our lab. PPR, pan-phage-resistant (resistant to all five phages used for training and to the entire 151 phage panel before training); PS, phage-susceptible (susceptible to all five phages used for training and altogether to 142/151 (94%) of the phage panel).

**Table 2:** Phages used in this work.

Phage	Propagation Strain	Genome Length, bp	Family	Genus	Host Range, %
KEN22	*Kp* MRSN 11382	166,645	*Straboviridae*	*Jiaodavirus*	26
KEN25-1	*Kp* MRSN 529046	169,768	*Straboviridae*	*Jiaodavirus*	19
KEN25-2	*Kp* MRSN 529046	165,574	*Straboviridae*	*Jiaodavirus*	23
KEN37	*Kp* MRSN 529046	166,503	*Straboviridae*	*Jiaodavirus*	34
KEN39	*Kp* MRSN 529046	166,254	*Straboviridae*	*Jiaodavirus*	36
KEN42	*Kp* MRSN 3619	38,200	*Autographiviridae*	*Teetrevirus*	18
KEN1821	*Kp* MRSN 529046	168,619	*Straboviridae*	*Jiaodavirus*	36
AFR4	*Kp* MRSN 3619	48,962	*Drexlerviridae*	*Webervirus*	17
EKq1	*Kq* MRSN 829456	48,244	Fmr. Siphoviridae *	*Unclass.*	15
EKq2	*Kq* MRSN 829456	51,496	*Drexlerviridae*	*Webervirus*	7

*Kp*, *K. pneumoniae*; *Kq*, *Klebsiella quasipneumoniae*. Phages KEN22, KEN25-1, KEN25-2, KEN37, and KEN39 [34] were used for training. Other phages were employed later for cocktail development. The genome of *K. quasipneumoniae* phage EKq1 was published [36]. All other phages have also been sequenced. The GenBank accession numbers for genome sequences of phages vB_Kpn3619-KEN42 (KEN42), vB_Kpn529046-KEN1821 (KEN1821, previously named KEN18-2-1), vB_Kpn3619-AFR4 (AFR4), and EKq2 are listed in the data availability statement below. * Unclassified phage EKq1 formerly belonged to the family *Siphoviridae* that is now excluded from the phage classification scheme.

**Table 3:** Evolved *K. pneumoniae* phages with altered or expanded host ranges.

Phage	Host Range	Expansion?
KEN22	26	Parental phage
KEN25-1	19	Parental phage
KEN25-2	23	Parental phage
KEN37	34	Parental phage
KEN39	36	Parental phage
15882-1	33	No
15882-3	44	Yes
15882-5	36	No
15882-6	40	Yes
15882-7	38	Yes
15882-8	14	No
27989-4	11	No
27989-11	9	No
27989-12	10	No

**Table 4:** Scoring system for phage plaque assay results.

Score	Observation
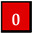	No activity, no lysis (negative result).
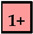	Lysis from without: very faint, turbid spots or clear spots in first dilutions, no plaque formation, and no negative dynamics of lysis; lysis, lysis, then nothing (negative result).
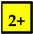	Clear or turbid spots, tiny plaques, countable or uncountable, or lack of visible isolated plaques but clear negative dynamics of lysis intensity from lower to higher dilution (slightly positive result).
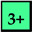	Clear spots, clear plaques of medium or small size (strictly positive result).
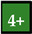	Totally clear spots, there are isolated large clear plaques in the highest phage dilutions (highly positive result).

**Table 5:** Comparison of *K. pneumoniae* phage cocktails WRAIR_KPM1 and WRAIR_KPM2.

Cocktail	Phage ID	Genome Size, bp	Family	Genus	Host Range	Mix Host Range
KPM1	AFR4	48,962	*Drexlerviridae*	*Webervirus*	17%	50%
	KEN39	166,254	*Straboviridae*	*Jiaodavirus*	36%	
	KEN42	38,200	*Autographiviridae*	*Teetrevirus*	18%	
	EKq1	48,244	*Fmr. Siphoviridae* *	*Unclass.*	15%	
	EKq2	51,496	*Drexlerviridae*	*Webervirus*	7%	
KPM2	AFR4	48,962	*Drexlerviridae*	*Webervirus*	17%	81%
	KEN39	166,254	*Straboviridae*	*Jiaodavirus*	36%	
	KEN42	38,200	*Autographiviridae*	*Teetrevirus*	18%	
	EKq1	48,244	*Fmr. Siphoviridae* *	*Unclass.*	15%	
	EKq2	51,496	*Drexlerviridae*	*Webervirus*	7%	
	KEN1821	168,619	*Straboviridae*	*Jiaodavirus*	36%	
	15882-3	167,537	*Straboviridae*	*Jiaodavirus*	44%	

* Unclassified phage EKq1 [36] formerly belonged to the family *Siphoviridae* now excluded from the phage classification scheme.

**Table 6:** Whole-genome nucleotide distance matrix based on multiple sequence alignment of five *Jiaodavirus* phages and four recombinant phages isolated from phage training.

	KEN22	KEN25-1	KEN25-2	KEN37	KEN39	15882-1	15882-3	15882-5	15882-6
KEN22		92.94	90.04	94.03	90.72	94.38	94.21	94.77	94.40
KEN25-1	92.94		87.70	92.12	89.44	90.17	90.23	90.52	90.19
KEN25-2	90.04	87.70		91.72	88.94	94.43	94.18	93.89	94.44
KEN37	94.03	92.12	91.72		91.33	92.94	93.07	92.54	92.97
KEN39	90.72	89.44	88.94	91.33		89.77	89.92	90.21	89.71
15882-1	94.38	90.17	94.43	92.94	89.77		99.34	99.15	99.65
15882-3	94.21	90.23	94.18	93.07	89.92	99.34		99.15	99.45
15882-5	94.77	90.52	93.89	92.54	90.21	99.15	99.15		99.27
15882-6	94.40	90.19	94.44	92.97	89.71	99.65	99.45	99.27	

## Data Availability

The BioProject number for this study is PRJNA1173206. The GenBank accession numbers for complete genome sequences of phages AFR4, EKq2, KEN1821, and KEN42 are PV240279, PV240280, PV240281, and PV240282, respectively. The GenBank accession numbers for genomes of evolved phages 15882-1, 15882-3, 15882-5, and 15882-6 are PQ537369, PQ537375, PQ537376, and PQ537382, respectively. The GenBank accession numbers for genomes of phage clones 15882-3-1 through 15882-3-5 and 15882-6-1 through 1588-6-5 from stability testing are PQ537370-PQ537374 and PQ537377-PQ537381, respectively.

## Supplementary Materials

The following supporting information can be downloaded at https://www.mdpi.com/article/10.3390/ijms26157597/s1.

## Abbreviations

The following abbreviations are used in this manuscript:

CDS	Protein coding sequence
HIB	Heart Infusion Broth
HIB-CM	Heart Infusion Broth with calcium chloride and magnesium sulfate
KPM	*Klebsiella* phage mix
LPS	Lipopolysaccharide
MDR	Multidrug-resistant
MIC	Minimum inhibitory concentration
MLST	Multilocus sequence typing
MRSN	Multidrug-Resistant Organism Repository and Surveillance Network
PPR	Pan-phage-resistant
PS	Phage-susceptible
SNP	Single-nucleotide polymorphism
ST	Sequence type
UTI	Urinary tract infection
WRAIR	Walter Reed Army Institute of Research
XDR	Extensively drug-resistant
XPR	Extensively phage-resistant
